# Supplementary material for: Root-Zone Restriction Regulates Soil Factors and Bacterial Community Assembly of Grapevine
Source: Int J Mol Sci. 2022 Dec 9;23(24):15628. doi: 10.3390/ijms232415628 (PMC9778885; doi:10.3390/ijms232415628)
Supplement: Supplementary file 1 [file ijms-23-15628-s001.zip › Figure Captions.pdf]

Figure Captions

Fig. S1

Taxonomic annotation of the bacterial community. longer the columns at the genus and species levels and the shorter at the phylum level, indicates a higher resolution of the annotation

Fig. S2

Alpha refraction curves of all the samples showing the observed OTU index. The flatness of the curve reflects the impact of sequencing depth on the diversity of the observed samples. The flatter the curve is, it indicates that the sequencing results are sufficient to reflect the diversity contained in the current samples

Fig. S3

Species accumulation curve reflect the rate of increase in new species observed as the sample size continues to expand over the course of sampling the population. Species accumulation curves are similar to rarefaction curves and are used to measure and predict the increase in species richness in a community with the expansion of the sample size

Fig. S4

Rank Abundance Curve shows the ASV ranks in each sample. The flatness of the broken line reflects the evenness of community composition. The gentler the broken line, the smaller the abundance difference between ASVs in the community, and the higher the evenness of the community composition. The steeper the broken line, the lower the evenness

Fig. S5

Unweighted pair-group method with arithmetic means (UPGMA) cluster analysis. The samples are clustered according to their similarity with each other on the basis of bray Curtis dissimilarity index. The shorter the branch length between the samples, the more similar the two samples are

Fig. S6

Canonical Correspondence analysis showing the correlation between the microbial communities of the sampling groups and the fruit quality indexes

Fig. S7

Graphical representation of the edaphic soil factors of the vineyard rhizosphere soil, showing the radical effects of root-zone restriction on the soil, at three phenological stages as Full bloom, veraison and maturity

Fig. S8

Network analysis on the basis of the Spearman Correlation between the edaphic factors and the bacterial community at genus level

Fig. S9

Flower petal diagram showing the unique and shared ASVs in the sampling Groups. Rhizophore soil(a), White roots(b), leaves(c), berries(d)

Fig. S10

Lefse Cladogram showing the bio-markers identified int eh bacterial community at different sampling stages (a). LDA score histogram showing the score of the identified microbial taxa, with a minimum lda score to quality was 2

Fig. S11

Top10 most abundant KEGG metabolic pathways int eh sampling groups are shown. Rhizophore soil(a), White roots(b), leaves(c), berries(d)

Fig. S12

Sub-network association with the sampling groups pie chart shown in the node. The pie-chart shows the contribution in terms of dominant taxa of sampling groups in a particular module. The Similarity index shows that the positive interactions are denoted by red lines while the negative with the green color. Rhizophore soil(a), White roots(b), leaves(c), berries(d)

Fig. S13

ZI-Pi scatter plot showing the network division in four major parts, peripherals, connectors, module hubs and network hubs. Rhizophore soil(a), White roots(b), leaves(c), berries(d)

Fig. S14

Subnetwork of dominant species with grouped abundance pie chart. The node (node) represents the ASV in the sample, and the size of the node is proportional to its abundance in (log2(CPM/n). The form of a pie chart shows the relative abundance ratio of the node in different samples (groups); the edge between nodes indicates that there is a correlation between the two connected nodes, the red line indicates a positive correlation, and the green line indicates a negative correlation

Text S1

PCR conditions and the library preparation protocols

Text S2

Network topological properties

Table S1

Statistic of sequencing volume per sample

Table S2

Relative abundance of the phylum

Table S3

Sub-network topologies list

Table S4

Node topologies list
