# Supplementary material for: Root-Zone Restriction Regulates Soil Factors and Bacterial Community Assembly of Grapevine
Source: Int J Mol Sci. 2022 Dec 9;23(24):15628. doi: 10.3390/ijms232415628 (PMC9778885; doi:10.3390/ijms232415628)
Supplement: Supplementary file 1 [file ijms-23-15628-s001.zip › Text S1.pdf]

Primers used in PCR and the optimal conditions:

PCR (polymerase chain reaction) amplification was performed with the bacterial V3V4-16S rRNA gene specific primers 338F(5'- *GCACCTA*ACTCCTACGGGAGGCAGCA-3') with a unique barcode sequence (italicized) and 806R (5'-GGACTACHVGGGTWTCTAAT-3') on following conditions: 98℃, 5 min (initial denaturation); 98℃, 10 s; 50℃, 30 s; 72℃, 30 s (for 25 cycles); 72℃, 5 min (final extension). PCR was conducted using Q5 high-fidelity DNA polymerase (NEB, M0491L) at 25 μl final volume with 20 ng template DNA, and the other PCR components are shown in the table below.

**Components**	**Quantity (μl)**
Q5 high-fidelity DNA polymerase	0.25
5*Reaction Buffer	5
5* High GC Buffer	5
dNTP Mix（10mM）	0.5
Template DNA	2
Former primer（10uM）	1
Reverse primer（10u）	1
ddH2O	10.25

Construction of metagenomic libraries:

The DNA Samples has a final volume of 50μl at 2 ng/μl。

DNA library preparation followed the manufacturer's instruction (Illumina). End repaired process adds 5'-phosphate groups needed for downstream ligation using an End Repair Mix with the following protocol.

(1) For each volume of 50.0μlDNA mixture add 10 μl of Control and 40μlof End Repaired Mix.

(2) Mix gently and incubate it on the pre-heated thermal cycler for 30 min at 30℃.

(3) Chean up the end repaired fragments with AMPure XP beads (Beckman, A63881), elute them in 15μl Resuspension Buffer.

In adenylate 3' ends process, a single ‘A’ nucleotide is added to the 3’ ends of the blunt fragments to make fragments compatible with adapters and prevent self-ligation. A corresponding single ‘T’ nucleotide on the 3’ end of the adapters provides a complementary overhang. Specific steps are as follows.

(1) Mix the following in a 30µl reaction volume: 15.0 µl of end repaired fragments, 2.5 µl of A-Tailing Control, and 12.5 of A-Tailing Mix.

(2) Mix gently and incubate it on the pre-heated thermal cycler for 30 min at 37℃.

Ligate adapters process to the ends of the DNA fragments, preparing them for hybridization onto a flow cell. In this process, The Ligation Mix joins adapters to fragments with the following protocol.

(1) For each 3’ end adenylated DNA sample, add 2.5 µl of Ligation Control, 2.5 µl of Ligation Mix and 2.5µl of the appropriate DNA adapter Index.

(2) Mix gently and incubate it on the pre-heated thermal cycler for 10 min at 30℃.

(3) Remove the samples from thermal cycler and add in 5.0 µl Stop Ligation Buffer in order to inactive the ligation mix.

After all processes above finished, DNA fragments that have adapter molecules on both sides should be enriched by PCR amplification. The program was 98℃, 30 s (initial denaturation); 98℃, 10 s; 60℃, 30 s; 72℃, 30 s (for 10 cycles); 72℃, 5 min(final extension). The pooled, adaptor-ligated, amplified library wa size-selected by running on 2% agarose gel electrophoresis in 1.0X TAE Buffer, then purified using the AxyPrep DNA Gel Extraction Kit (Axygen, xxx). Analyze 1 µL of amplified library on the Agilent® Bioanalyzer™ instrument with the Agilent High Sensitivity DNA Kit (Cat. no. 5067-4626). And quantified on Promega® QuantiFluor™ with Quant-iT PicoGreen dsDNA Assay Kit (Invitrogen, P7589).

3. Sequencing

We performed mate-pair sequencing on 2*300 base pairs (bp) with MiSeq Reagent Kit v3(600-cycles-PE) (Illumina, MS-102-3003) for the library on Miseq. The library with fixed adaptors is denatured to single strands and grafted to the flow cell, followed by bridge amplification to form clusters which contains clonal DNA fragments. Four kinds of nucleotides (ddATP, ddGTP, ddCTP, ddTTP) which contain different cleavable fluorescent dye and a removable blocking group would complement the template one base at a time, and the signal could be captured by a (charge-coupled device) CCD. MiSeq uses two lasers and four filters to detect four types of nucleotide (A, T, G, and C). MiSeq control system (MCS v2.4.1) and real-time analyzer (RTA) is in charge of picture background normalization, signal location correction, cross-talk correction, signals conversion, and sequencing data generation.
