# Supplementary material for: Root-Zone Restriction Regulates Soil Factors and Bacterial Community Assembly of Grapevine
Source: Int J Mol Sci. 2022 Dec 9;23(24):15628. doi: 10.3390/ijms232415628 (PMC9778885; doi:10.3390/ijms232415628)
Supplement: Supplementary file 1 [file ijms-23-15628-s001.zip › Text S2.pdf]

topo	values
RMT_threshold	0.74
Average_nearest_neighbor_degree	25.01333782
Average_path_length	3.511453859
Betweenness_centrality	420738.7212
Closeness_centrality	0.676628189
Degree_assortativity	0.293392533
Degree_centralization	15104
Density	0.0688248
Cluster_num	12
Diameter	9.269215492
Transitivity	0.660557491
Num_vertice	242
Num_edge	2007
Modularity	0.296098057

Network topologies for soil

topo	values
RMT_threshold	0.74
Average_nearest_neighbor_degree	11.79498297
Average_path_length	4.963187729
Betweenness_centrality	1653704.012
Closeness_centrality	0.458588853
Degree_assortativity	0.425093282
Degree_centralization	7482
Density	0.031078311
Cluster_num	16
Diameter	11.82842911
Transitivity	0.52500812
Num_vertice	271
Num_edge	1137
Modularity	0.649906461

Network topologies for white roots

Topo	values
RMT_threshold	0.74
Average_nearest_neighbor_degree	12.24970549
Average_path_length	3.970492381
Betweenness_centrality	571029.6708
Closeness_centrality	0.2911707
Degree_assortativity	0.458986028
Degree_centralization	6522
Density	0.034885001
Cluster_num	11
Diameter	8.593964637
Transitivity	0.513361822
Num_vertice	234
Num_edge	951
Modularity	0.561135882

Network topologies for leaves

topo	empirical_network
RMT_threshold	0.74
Average_nearest_neighbor_degree	10.95472021
Average_path_length	4.115559187
Betweenness_centrality	495713.9084
Closeness_centrality	0.277691431
Degree_assortativity	0.518415646
Degree_centralization	7026
Density	0.030709983
Cluster_num	15
Diameter	10.09894782
Transitivity	0.517667375
Num_vertice	247
Num_edge	933
Modularity	0.561028843

Network topologies for berries
