# Supplementary figures and images for: Root-Zone Restriction Regulates Soil Factors and Bacterial Community Assembly of Grapevine
Source: Int J Mol Sci. 2022 Dec 9;23(24):15628. doi: 10.3390/ijms232415628 (PMC9778885; doi:10.3390/ijms232415628)

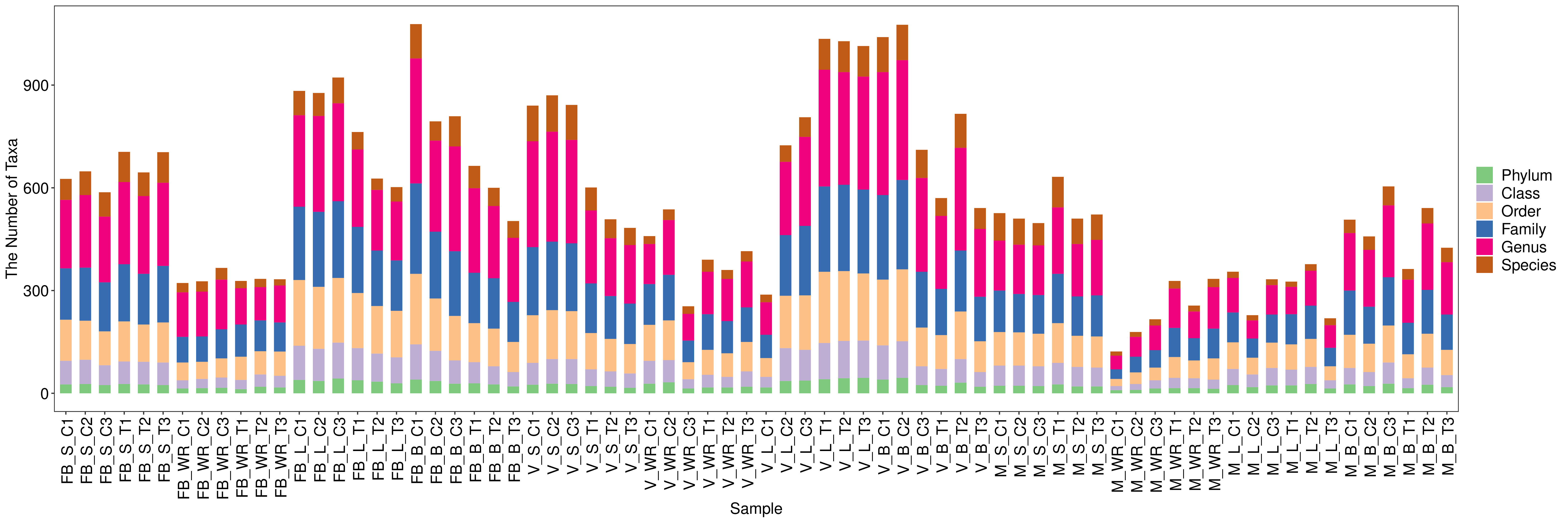

Supplement: Supplementary file 1 [file ijms-23-15628-s001.zip › Fig. S1.png]

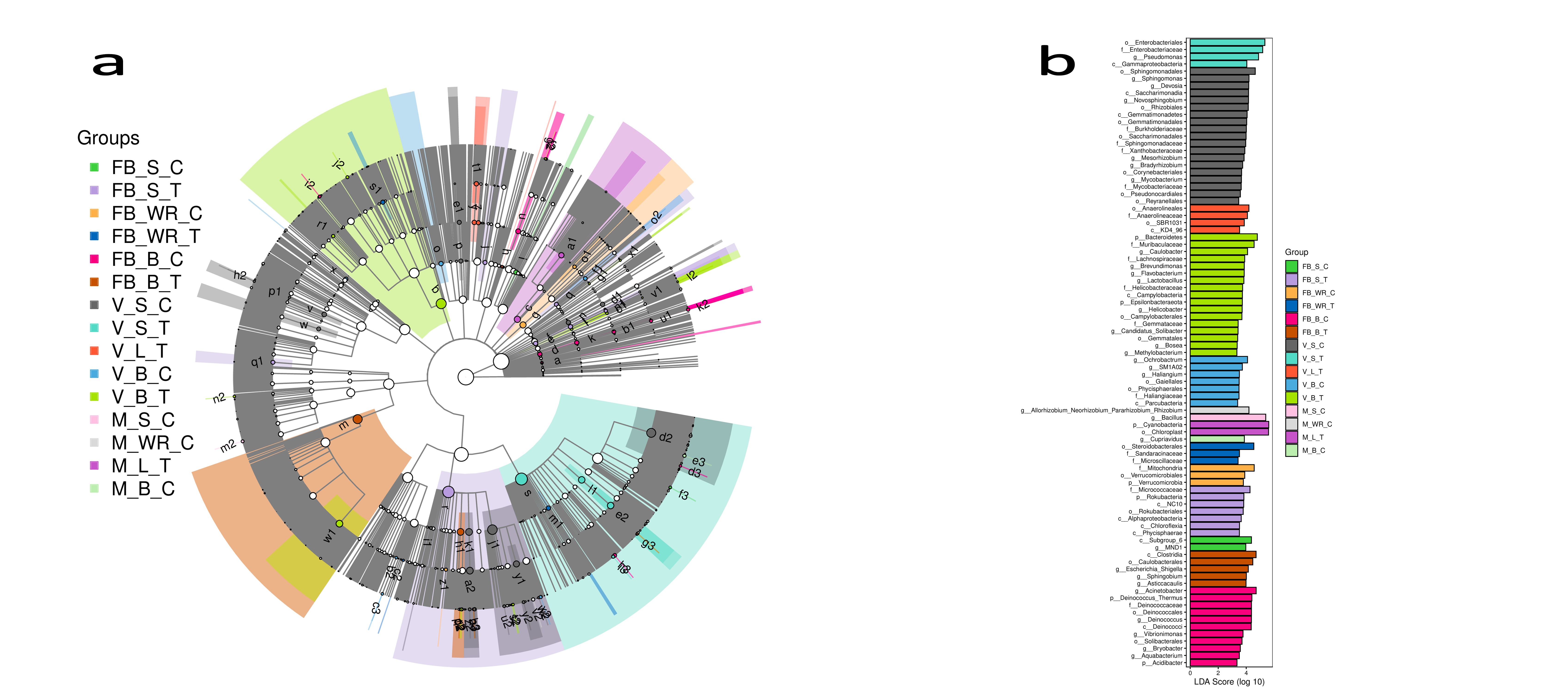

Supplement: Supplementary file 1 [file ijms-23-15628-s001.zip › Fig. S10.png]

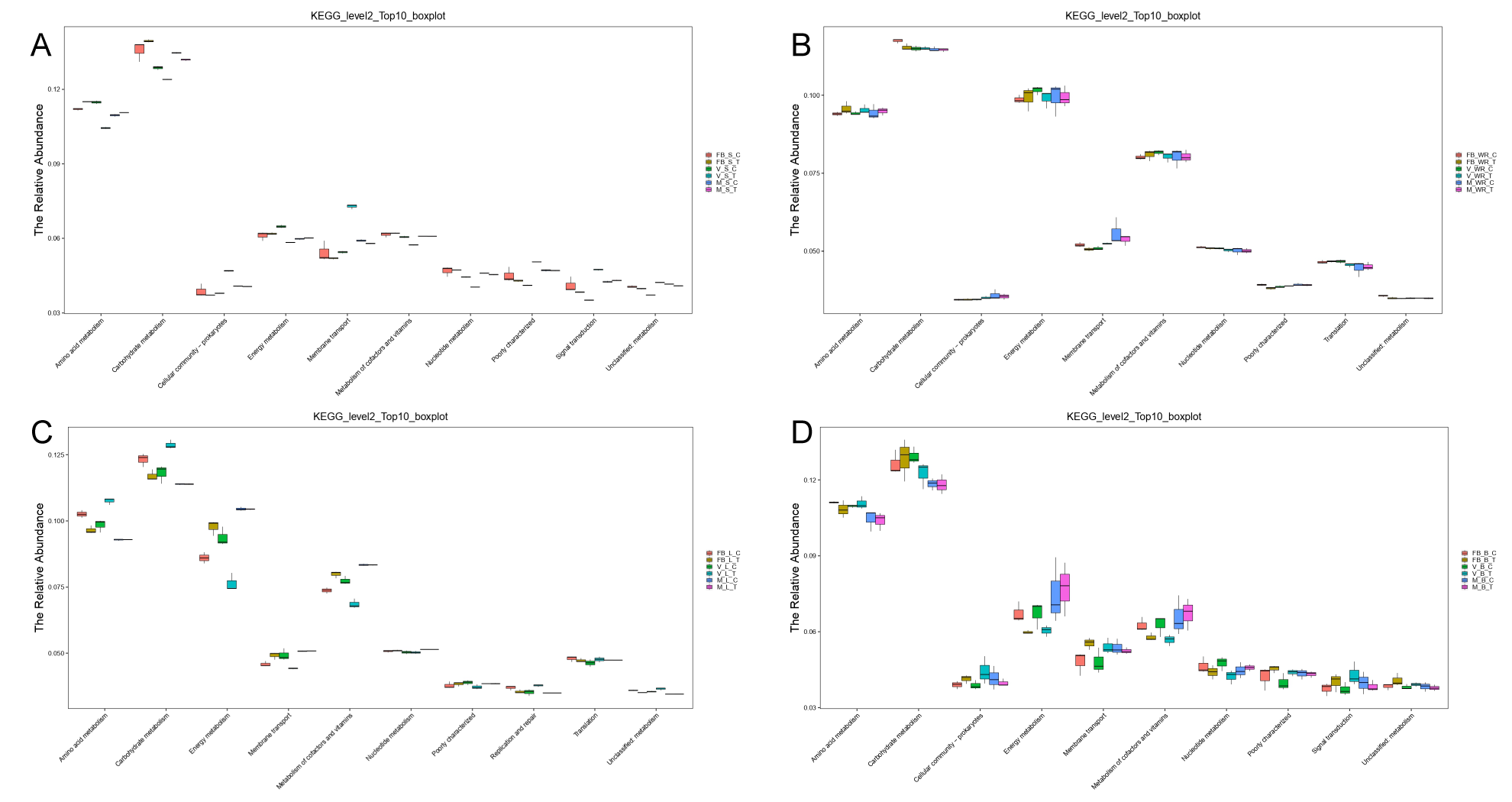

Supplement: Supplementary file 1 [file ijms-23-15628-s001.zip › Fig. S11.png]

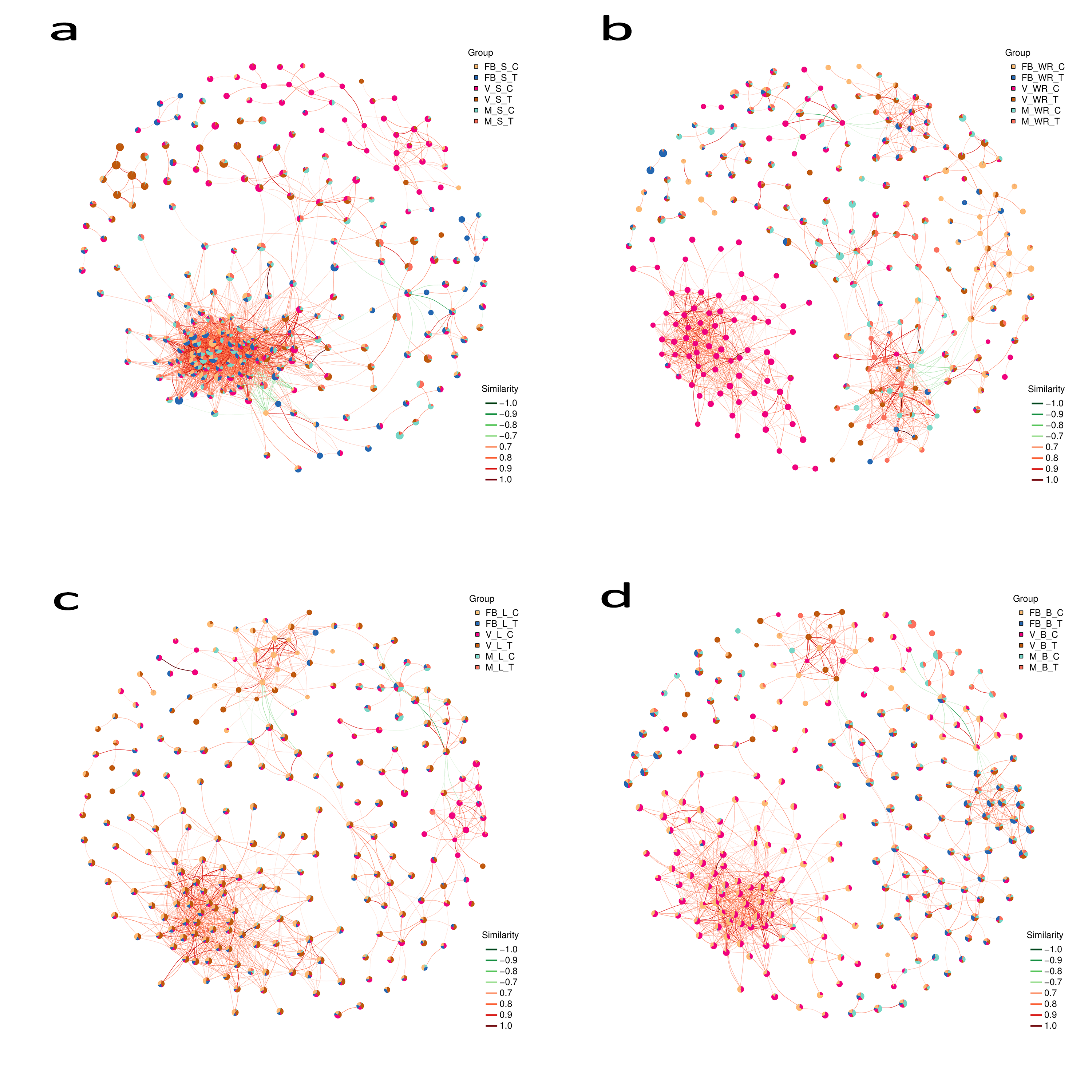

Supplement: Supplementary file 1 [file ijms-23-15628-s001.zip › Fig. S12.png]

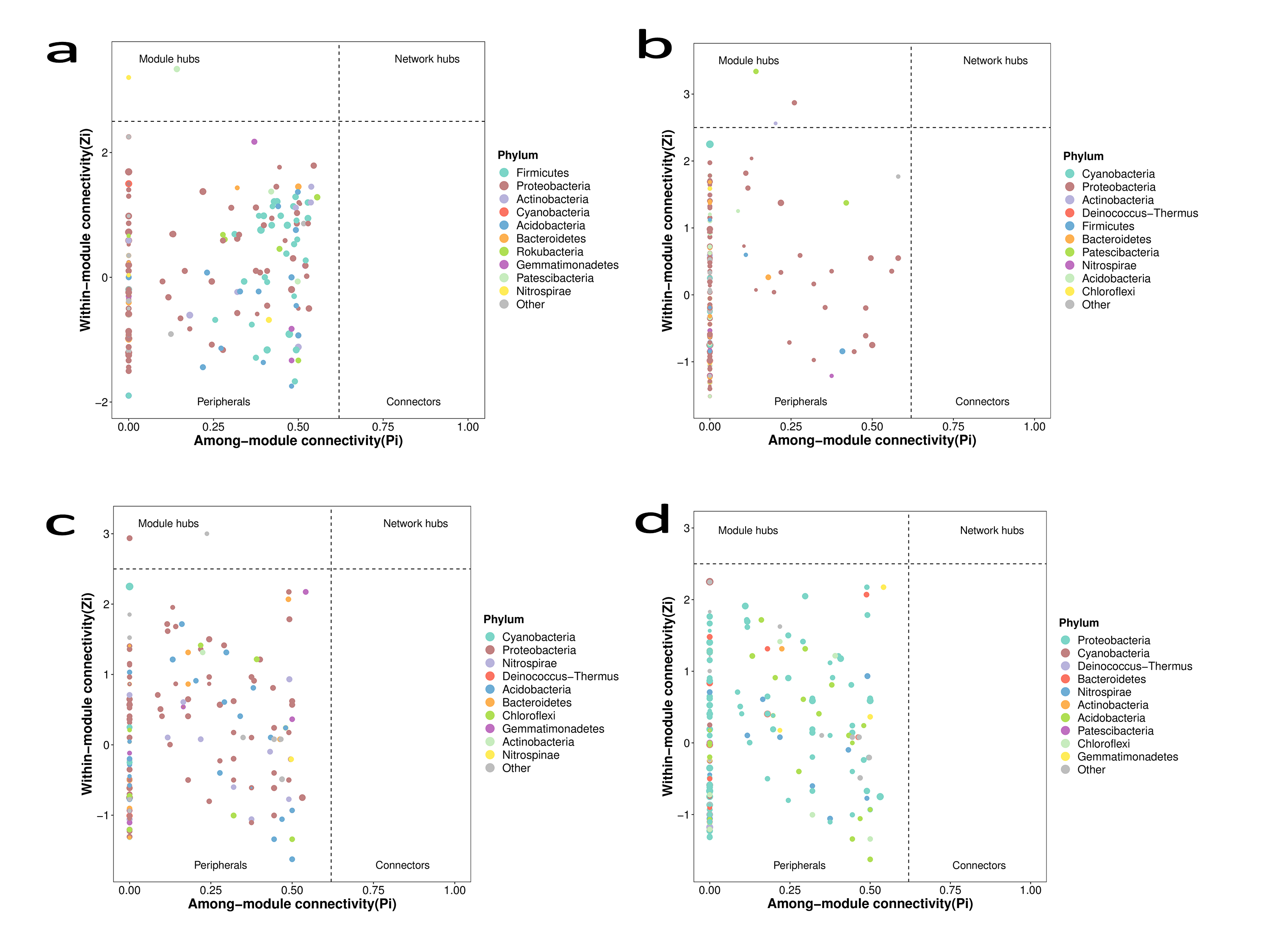

Supplement: Supplementary file 1 [file ijms-23-15628-s001.zip › Fig. S13.png]

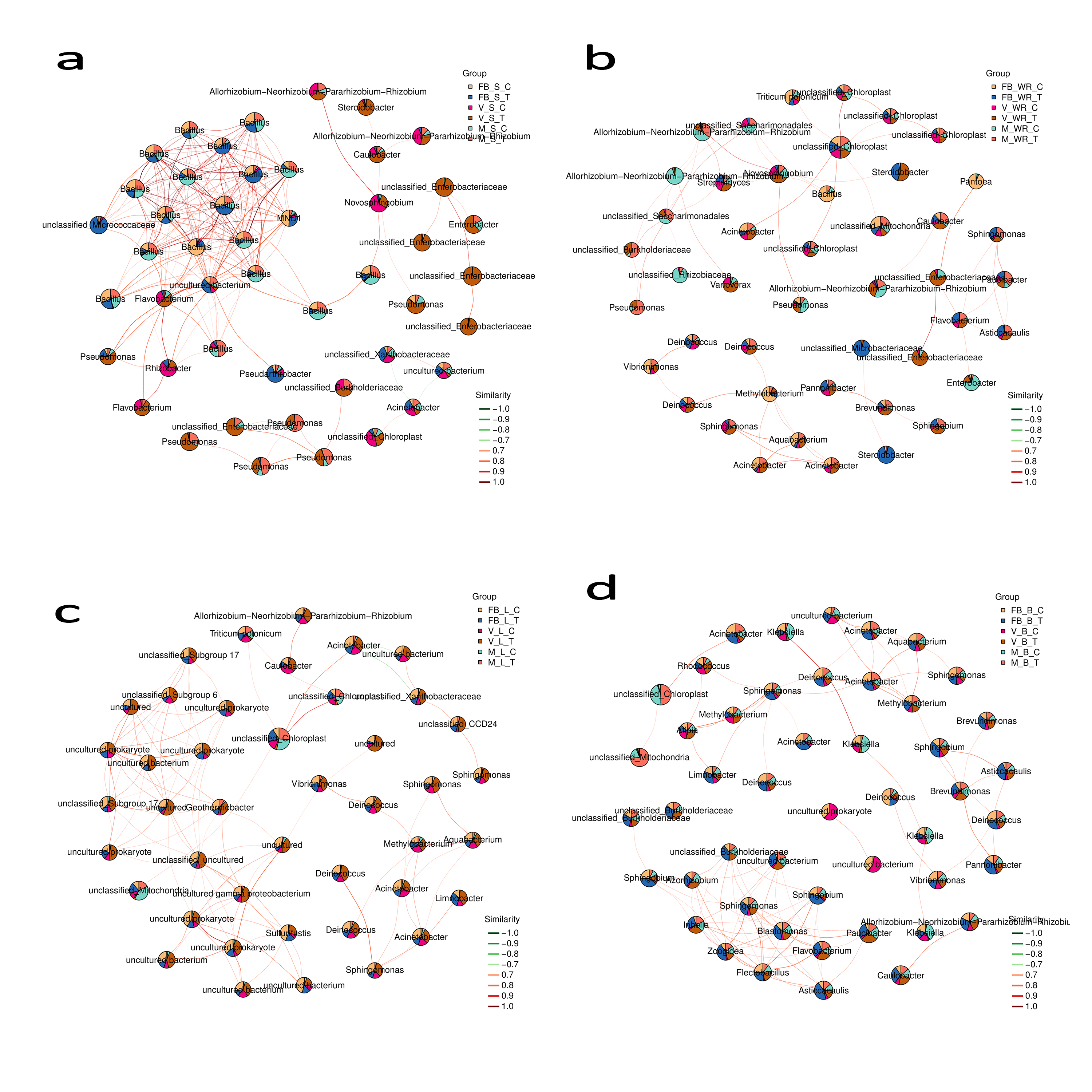

Supplement: Supplementary file 1 [file ijms-23-15628-s001.zip › Fig. S14.png]

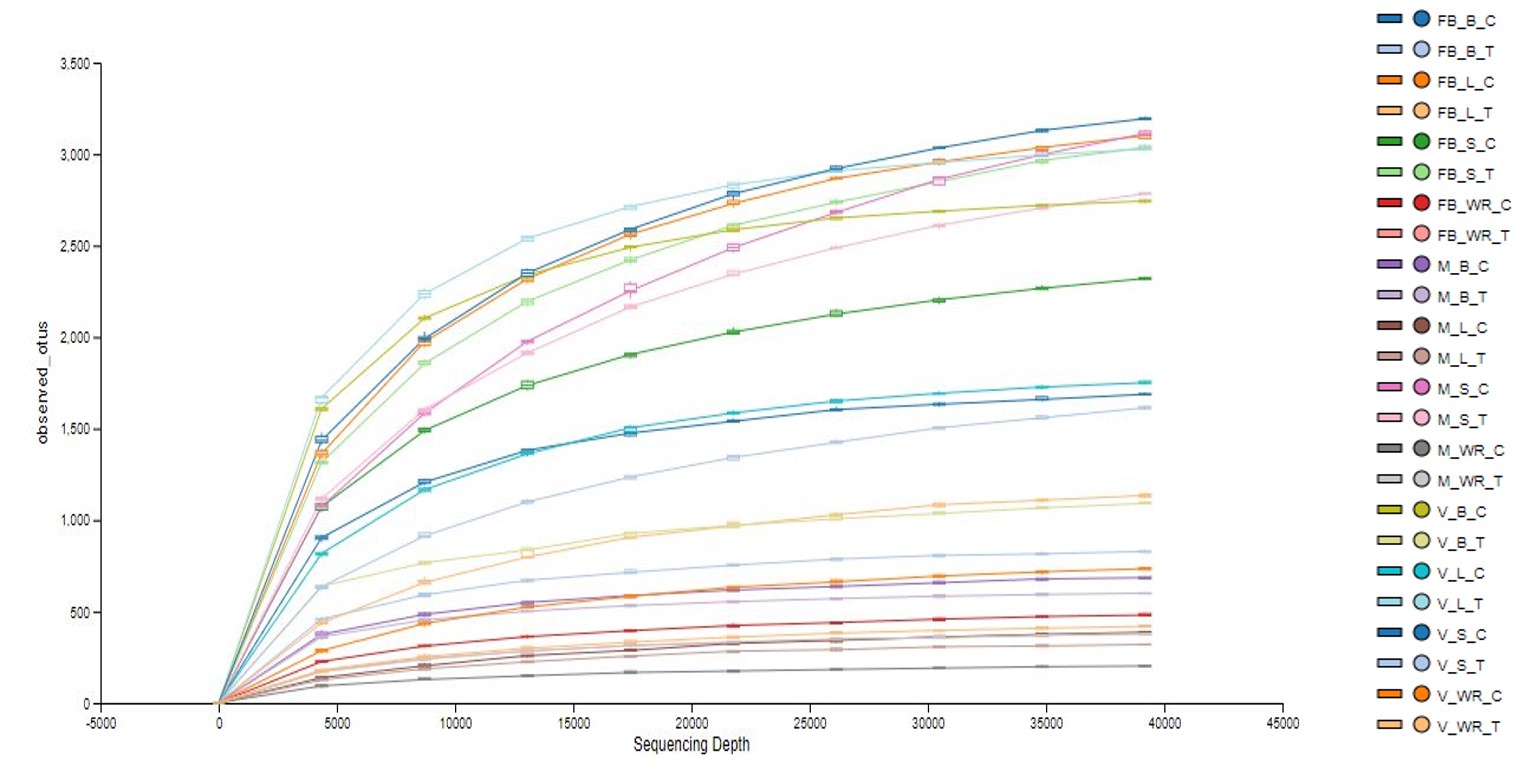

Supplement: Supplementary file 1 [file ijms-23-15628-s001.zip › Fig. S2.JPG]

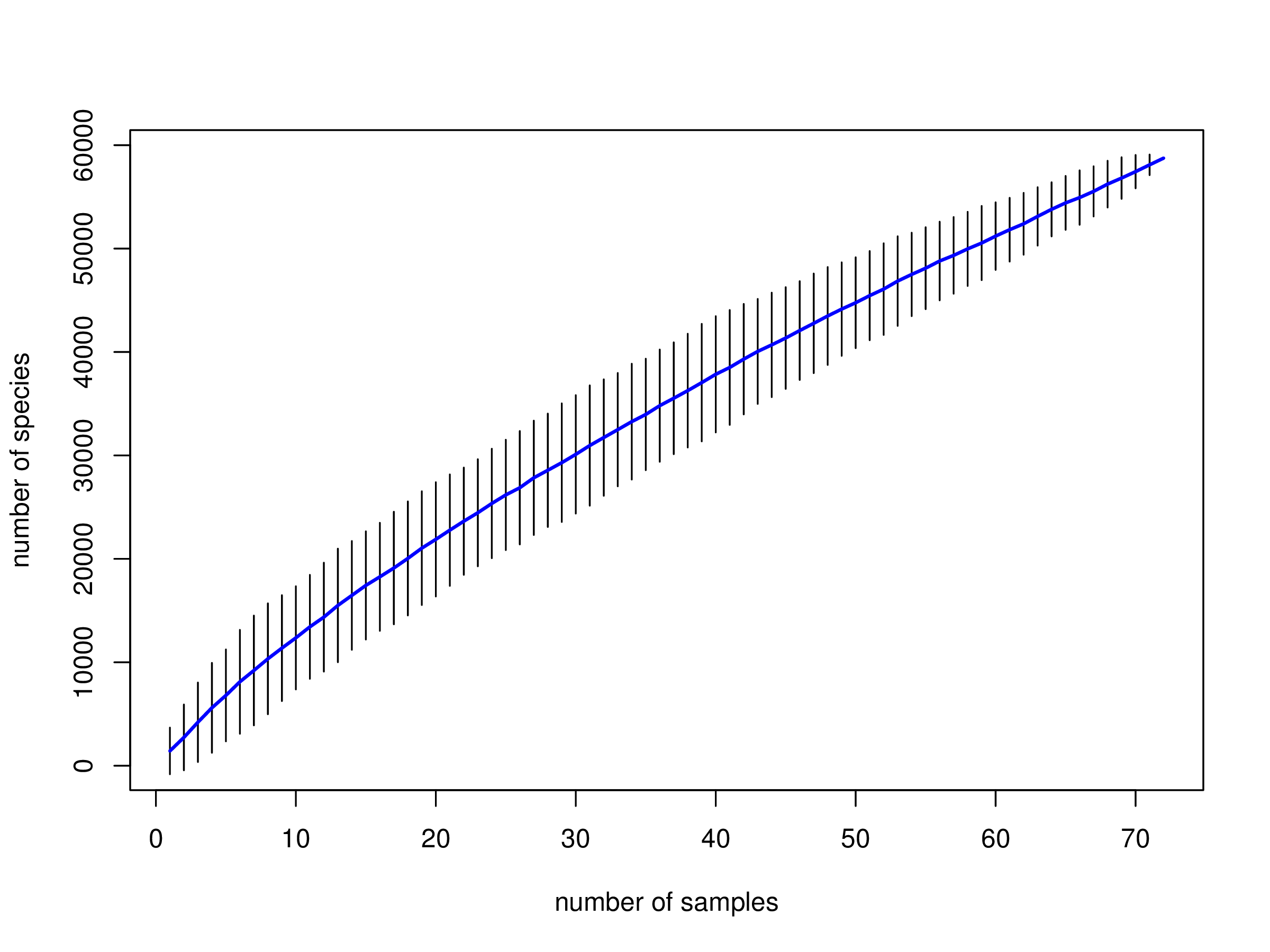

Supplement: Supplementary file 1 [file ijms-23-15628-s001.zip › Fig. S3.png]

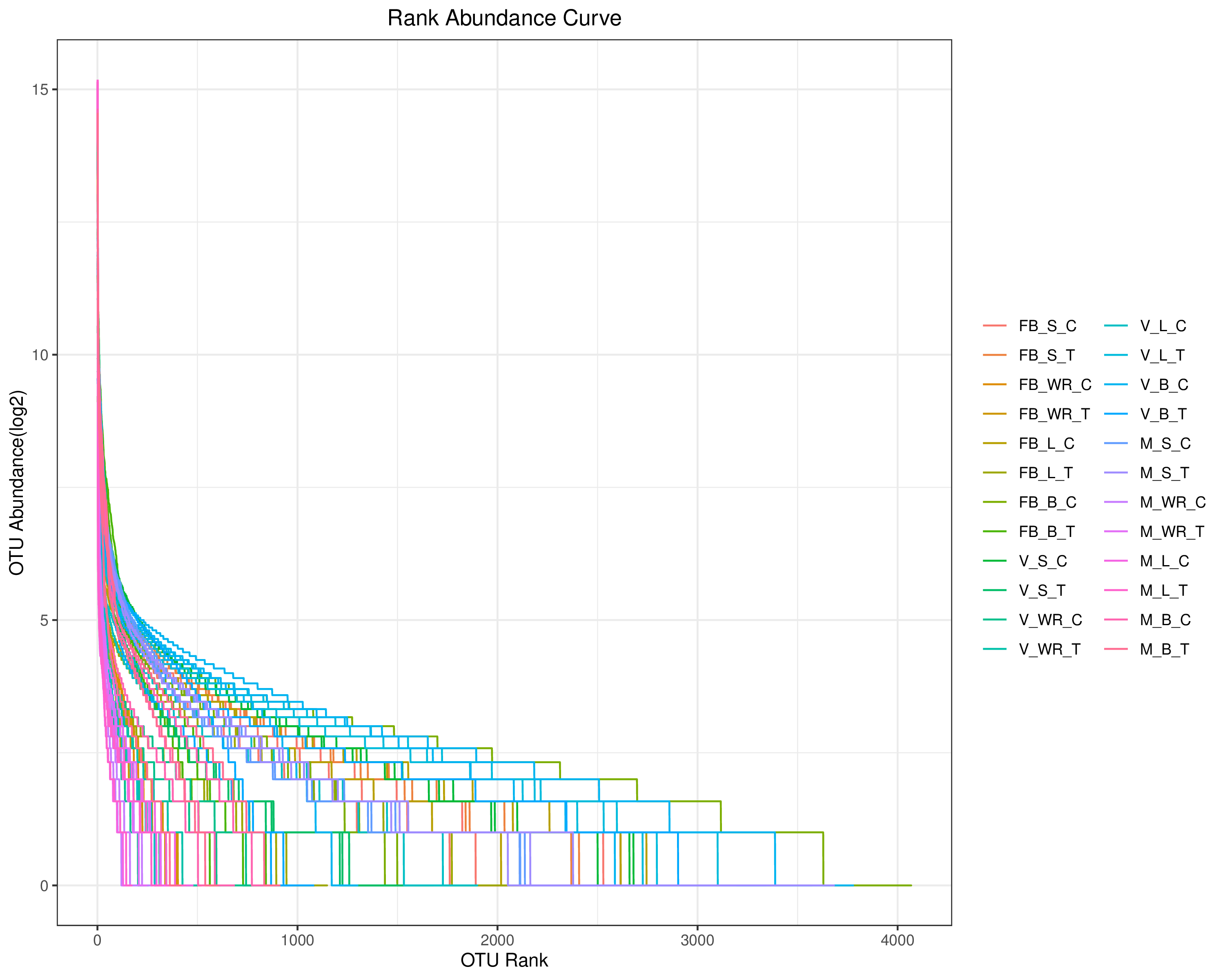

Supplement: Supplementary file 1 [file ijms-23-15628-s001.zip › Fig. S4.png]

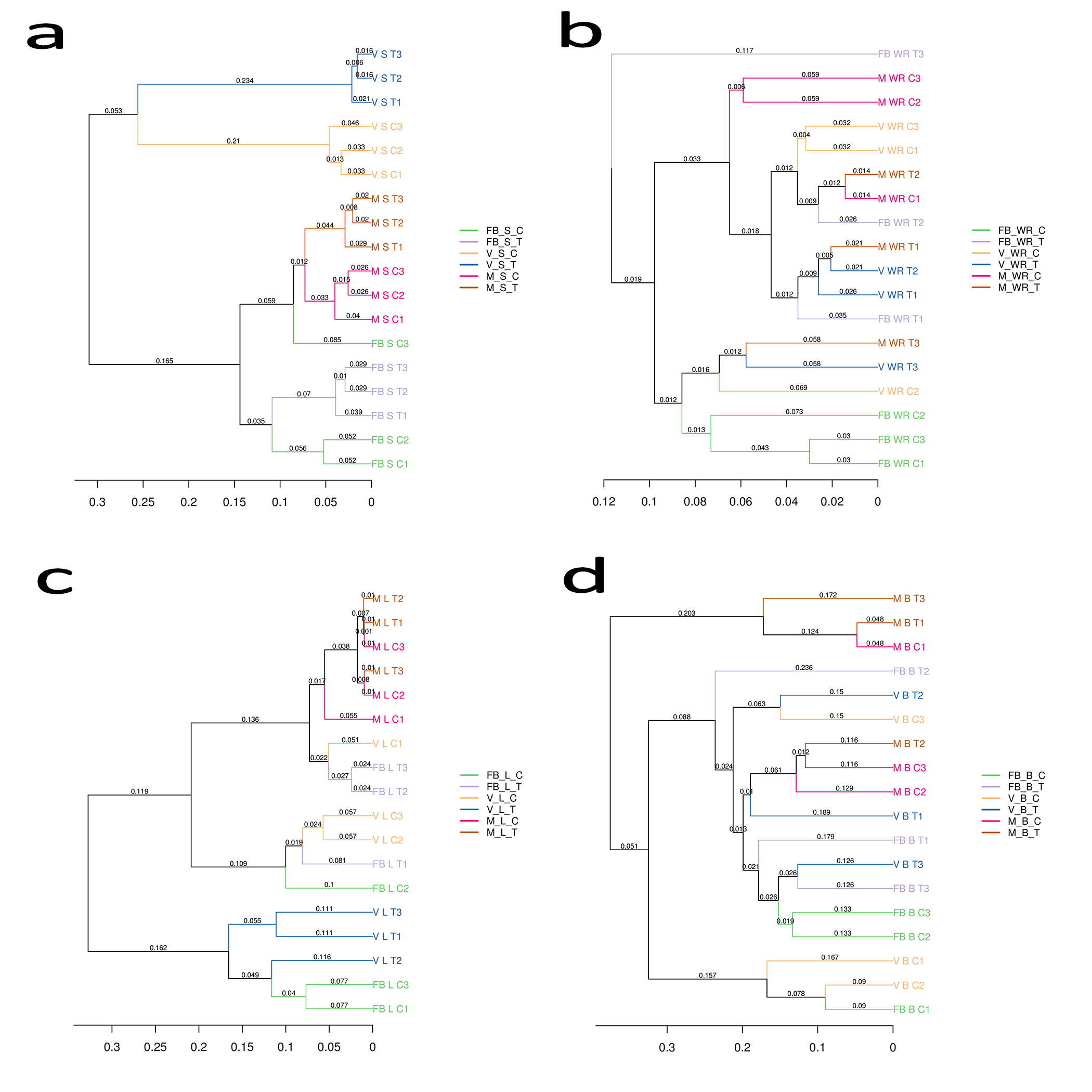

Supplement: Supplementary file 1 [file ijms-23-15628-s001.zip › Fig. S5.png]

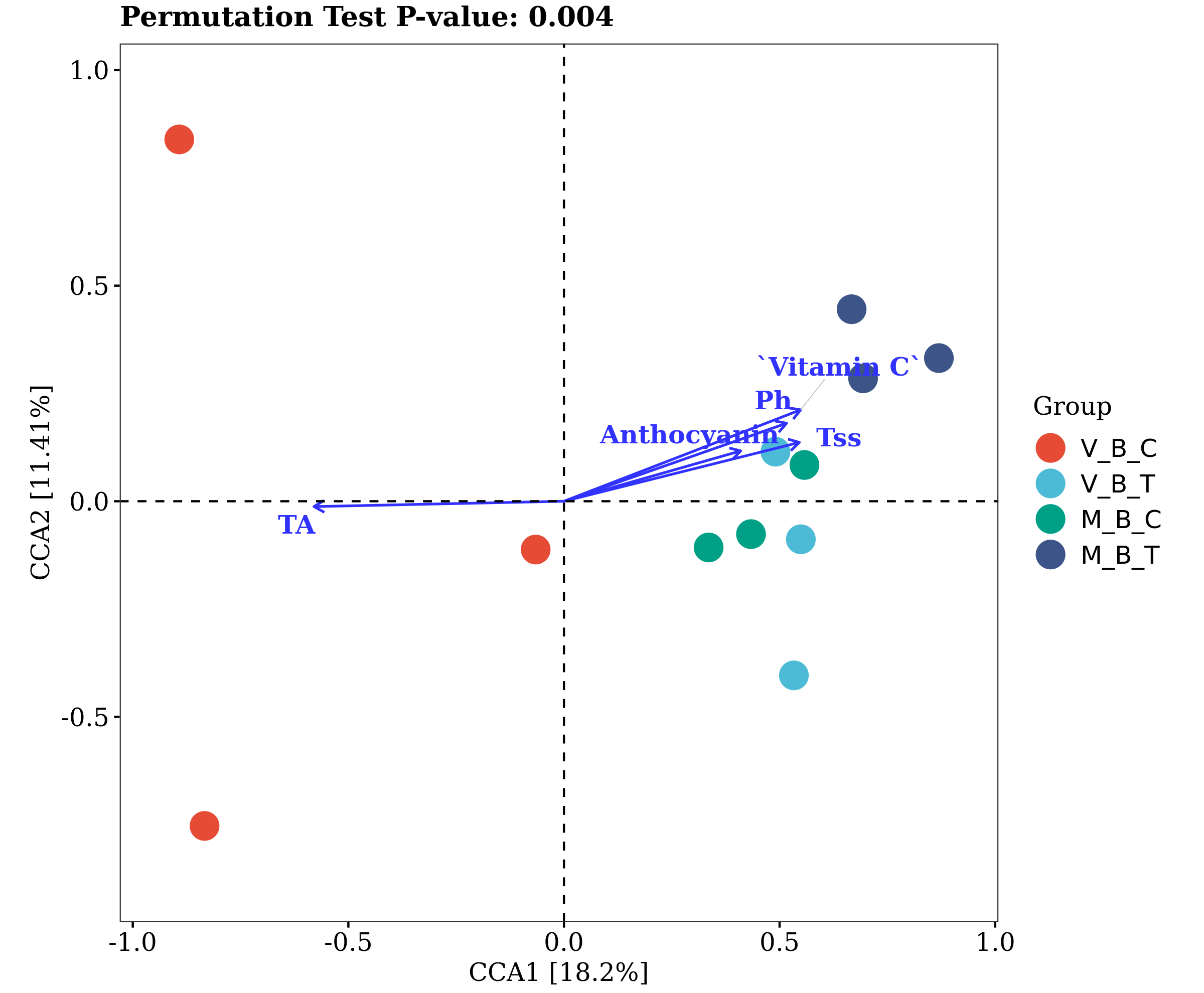

Supplement: Supplementary file 1 [file ijms-23-15628-s001.zip › Fig. S6.png]

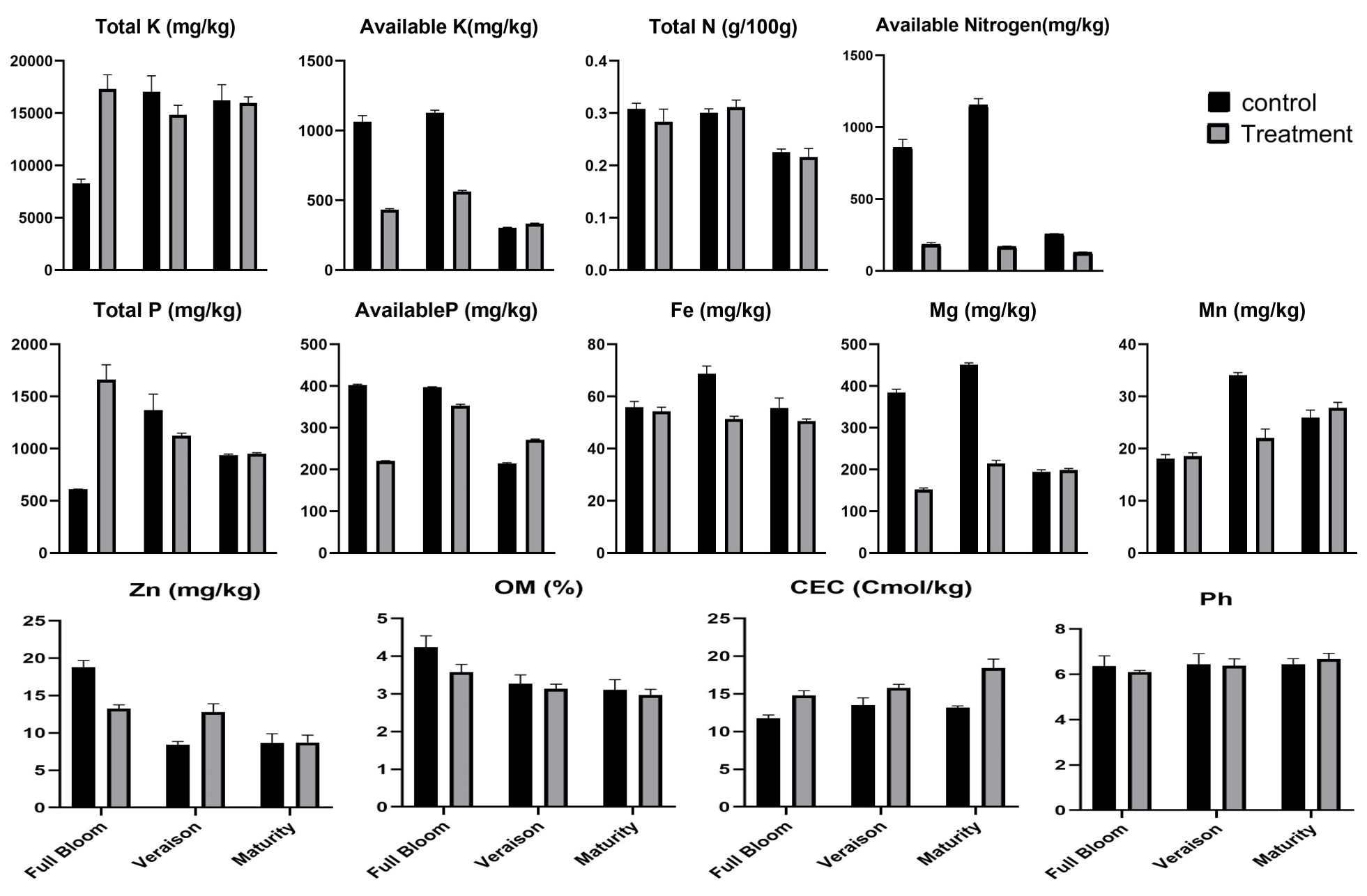

Supplement: Supplementary file 1 [file ijms-23-15628-s001.zip › Fig. S7.jpg]

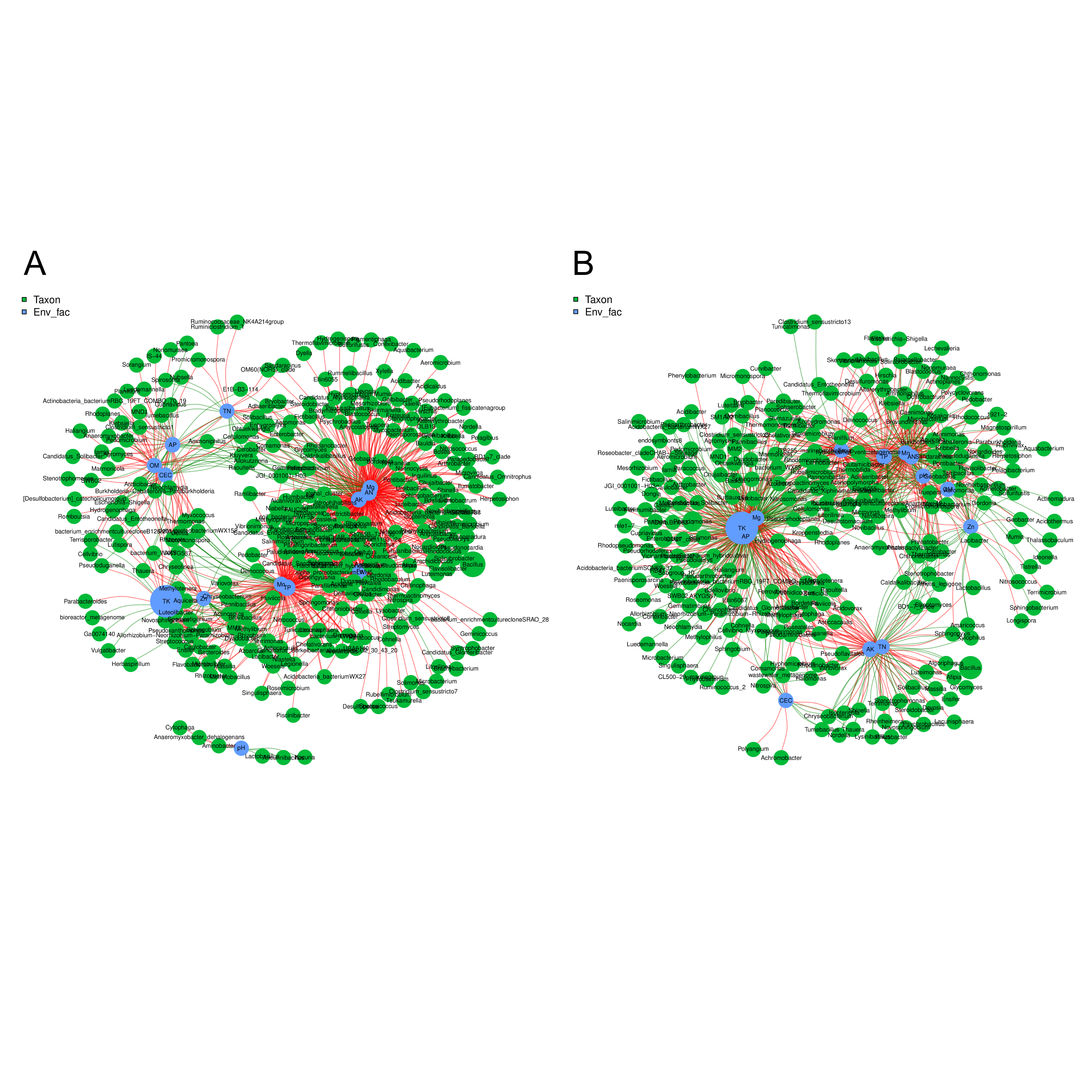

Supplement: Supplementary file 1 [file ijms-23-15628-s001.zip › Fig. S8.png]

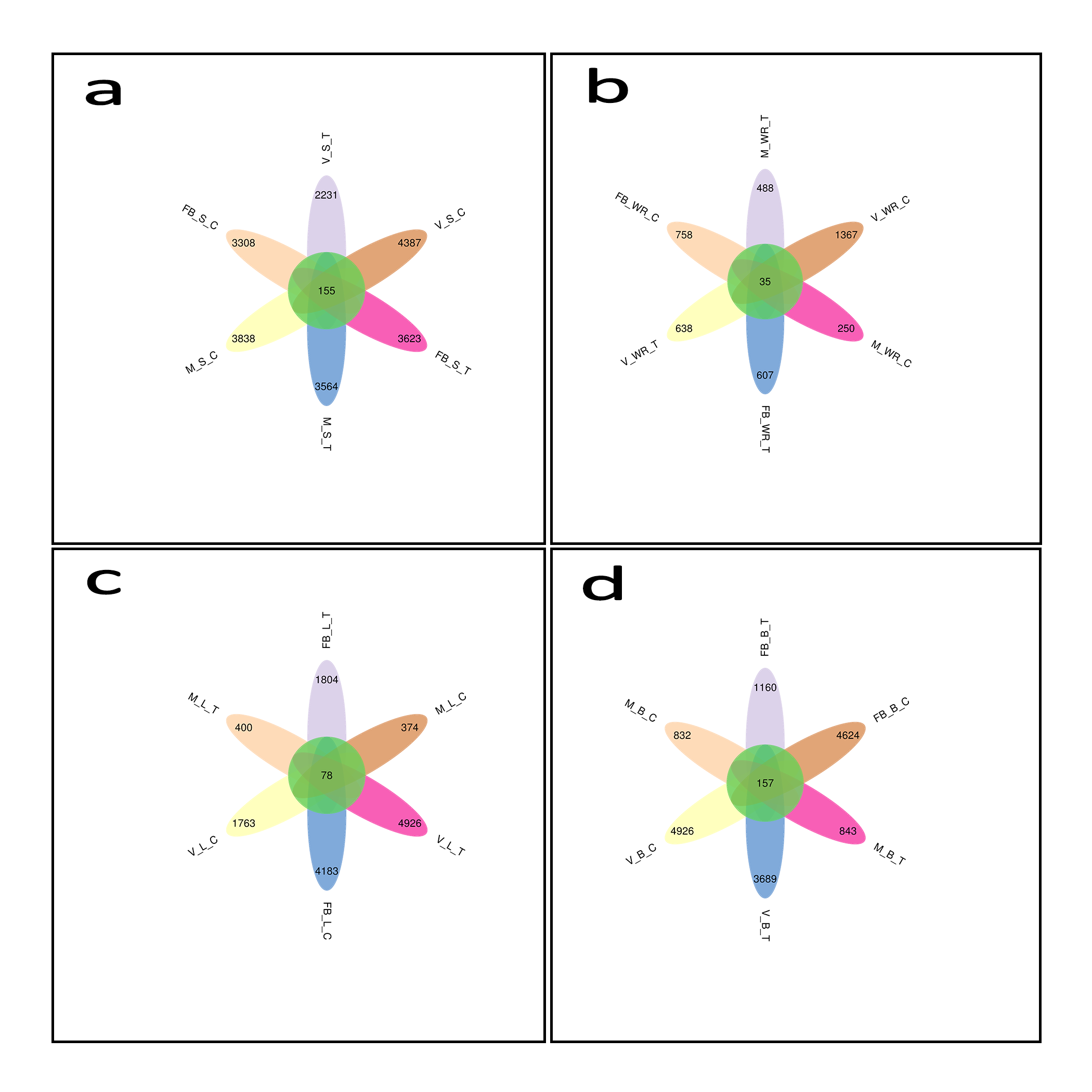

Supplement: Supplementary file 1 [file ijms-23-15628-s001.zip › Fig. S9.png]
